# Brain Mass and Cranial Nerve Size in Shrews and Moles

**DOI:** 10.1038/srep06241

**Published:** 2014-09-01

**Authors:** Duncan B. Leitch, Diana K. Sarko, Kenneth C. Catania

**Affiliations:** 1 Department of Biological Sciences, Vanderbilt University, Nashville, TN, USA; 2 Department of Anatomy, Cell Biology & Physiology, Edward Via College of Osteopathic Medicine, Spartanburg, SC, USA

## Abstract

We investigated the relationship between body size, brain size, and fibers in selected cranial nerves in shrews and moles. Species include tiny masked shrews (*S. cinereus*) weighing only a few grams and much larger mole species weighing up to 90 grams. It also includes closely related species with very different sensory specializations – such as the star-nosed mole and the common, eastern mole. We found that moles and shrews have tiny optic nerves with fiber counts not correlated with body or brain size. Auditory nerves were similarly small but increased in fiber number with increasing brain and body size. Trigeminal nerve number was by far the largest and also increased with increasing brain and body size. The star-nosed mole was an outlier, with more than twice the number of trigeminal nerve fibers than any other species. Despite this hypertrophied cranial nerve, star-nosed mole brains were not larger than predicted from body size, suggesting that magnification of their somatosensory systems does not result in greater overall CNS size.

There is a long history of interest in the relationship between brain size and body size and how variations in brain size may relate to intelligence. Brain size is known to correlate highly with body size with correlation coefficients, including encephalization quotients, which are typically greater than 0.90 in various studies^1^,^2^,^3^. Thus for species within a given phylogenetic group there is a predicted brain size for a given body size.

When ratios of brain size to body size are considered, it is often assumed that brain weight is a surrogate for some underlying, unknown variable that scales with body size and is required for maintaining a basic behavioral repertoire^4^,^5^,^6^,^7^. One possibility is that sensory inputs and motor outputs scale at some rate with body size and that brains must increase in size to handle these larger volumes of afferents and efferents. Extra computational ability, or simply put - intelligence, is thought to accompany brains that are larger than needed for the “house-keeping” tasks of a given body size. Yet we have very little direct evidence for how afferents and efferents scale with brain and body size (but see References 6 through 8). On the other hand, increased “information processing capabilities,” a concept that has been proposed as a correlate to intelligence^8^, may occur without changes to relative brain mass. In this case, it likely that organizational changes to the cortex and CNS underscore the increased sensory processing rather than more generalized changes to the absolute size of the CNS^9^.

In addition to influences of body size on brain size and fiber tracts, elaboration of particular senses could be correlated with changes in the number of sensory inputs and perhaps brain size. A number of specialized sensory systems have been investigated at the level of representation in the central nervous system and sensory periphery. For example the number of fibers supplying rodent whiskers has been compared to the central representation of whiskers (barrels) in sensory maps^10^,^11^. However, anatomical changes to the relays between the sensory or sensorimotor periphery and the CNS have not been examined in as great detail (see Ref. 12 for quantitative optic nerve comparisons among diurnal and nocturnal primates).

In light of previous investigations of CNS adaptation found in the star-nosed mole (*Condylura cristata*) which have focused on the magnification within primary and secondary somatosensory cortex^13^, principal trigeminal nucleus^14^, and spinal trigeminal subnuclei^15^ this tactile specialist offers the chance to compare potential changes of overall brain size as a possible consequence of increased sensory input. For example it is possible that natural selection has produced a larger than expected brain corresponding to the large amount of CNS devoted to the nasal representation. This would be in keeping with investigations of a wide range of mammals of different niches and brain sizes across developmental time, indicating that enlargement of the entire brain (minus the olfactory system) is associated with selection for particular behavioral abilities^16^. These findings suggest a “simple rule” concerning the volume of particular areas of the brain increasing in a regular, predictable manner over development, with elaborations to particular areas occurring as consequence of alterations to the timing and duration of isocortical neurogenesis, ultimately resulting in a larger brain^4^,^17^.

On the other hand specialization of a particular area of the central nervous system might take place independent of brain size. For example, increases to one area of the brain might occur at the expense of adjacent areas. Across the isocortex, this appears to be the case when examining primary sensory organization of the subterranean rodent mole-rat species *Heterocephalus glaber* which shows a greatly enlarged somatosensory cortex and tiny visual cortex (in comparison to rats)^18^, presumably reflecting the selection pressures for tactile information over information transmitted through the atrophied visual system^19^. More globally, the size of the entire brain has been interpreted as a competitive process of tradeoff for costly neural tissue in so-called “push-pull” relationships; this is suggested to be the case with reductions in the limbic and olfactory systems as related to increased primate visual dependence^20^. Whether or not this is the situation among the small-brained insectivore species, especially those of featuring elaborate sensory specialization, is not known.

Here we examine scaling of brain and body mass and the potential influence of changes in the sensory periphery through counts of afferents of the trigeminal nerve (CN V) which mediates somatosensory information from the head and face, the cochlear component of the vestibulocochlear nerve (CN VIII) which directs audition, and the optic nerve (CN II) which is responsible for visual input. These results were compared to measures of body size and brain size in seven species within a group of closely related small mammals historically classified as insectivores (shrews – Family Soricidae, and moles - Family Talpidae). The species ranged in size from the small 4 gram masked shrew (*Sorex cinereus*) to the larger, 91 gram eastern American mole (*Scalopus aquaticus*). These results regarding the scaling of brains, bodies, and fibers tracts with body size are briefly discussed in the context of other mammals.

## Results

**Figure 1:**
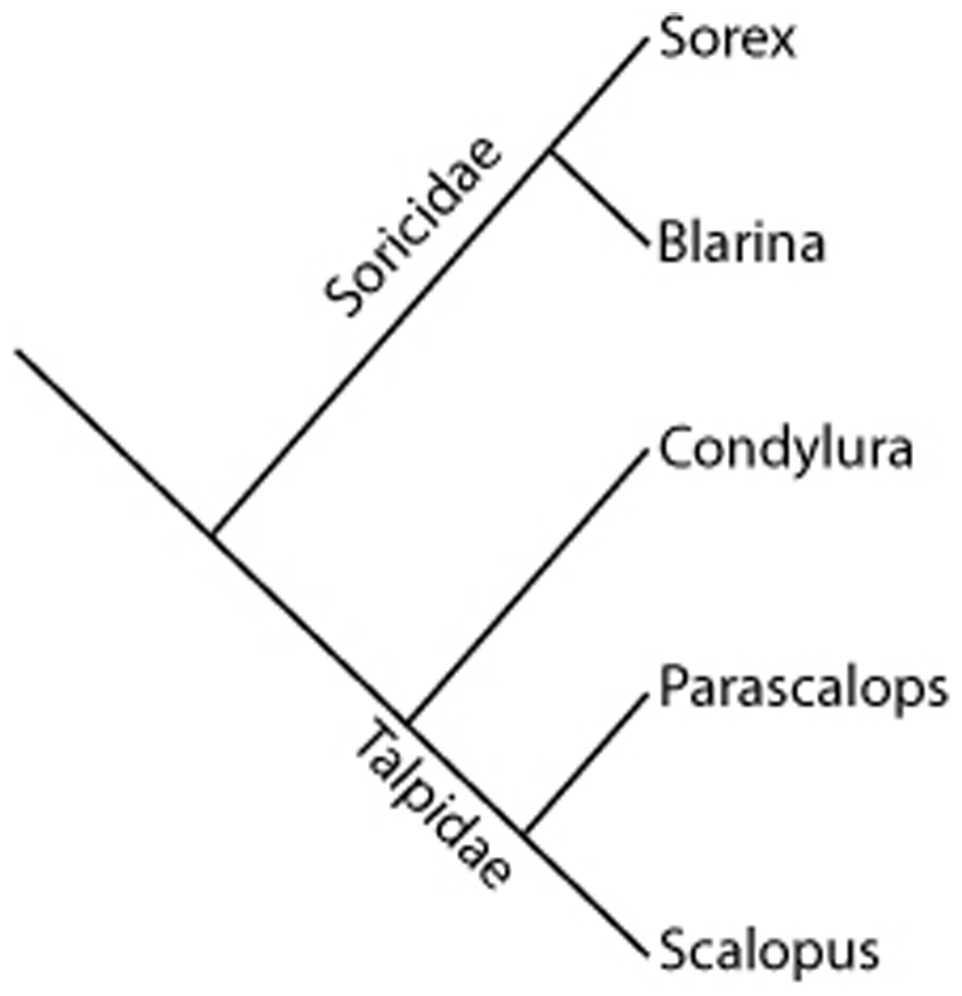
Schematic of phylogeny of “Insectivora” (Eulipotyphyla) species used in this investigation. The shrews are in the family Soricidae and are represented in the Sorex and Blarina genera. Their sister group is the family Talpidae, with examples from Condylura, Parascalops, and Scalopus genera. Adapted from Grenyer and Purvis, 2003 and Symonds, 2005.

**Figure 2:**
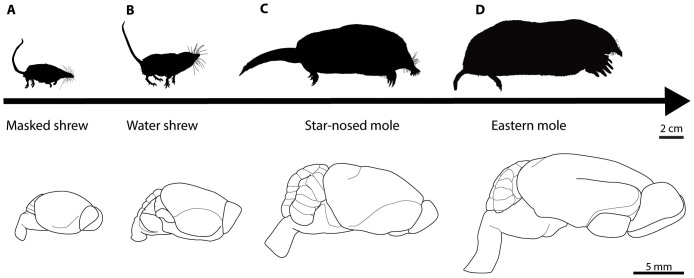
Body and brain scaled size comparisons among select species. Profiles of the body shape and size of the (A) masked shrew, (B) water shrew, (C) star-nosed mole, and (D) eastern mole illustrate the more than 36-fold change in body mass (masked shrew to eastern mole) among the examined animals. Despite overall similarities in gross morphology (large olfactory bulbs, lissencephalic forebrains, and prominent cerebellums), the brains of these species vary in length from about 1 cm in the masked shrew and the water shrew to about 1.7 cm in the star-nosed mole and 2.5 cm in the eastern mole. This figure was drawn by D.B.L.

Shrews and moles are part of the monophyletic order Eulipotyphyla and represent closely related groups as shown in Figure 1 (adapted from^21^,^22^). For each species, the brain mass, body mass, olfactory bulb mass, and number of axons within selected sensory cranial nerves were measured. Some nerve tracts could not be confidently identified in some species and were not included (e.g., the optic nerve of the eastern mole). The shrews used in this analysis ranged in total body mass from approximately 3.8 grams (*S. cinereus*) to 16 g (*S. palustris*) (Table S1 and Fig. 2A, B) whereas the mole body sizes ranged from roughly 50 grams for the smaller *P. breweri* and *C. cristata* to 90 grams for *S. aquaticus* (Table S1 and Fig. 2C, D).

**Figure 3:**
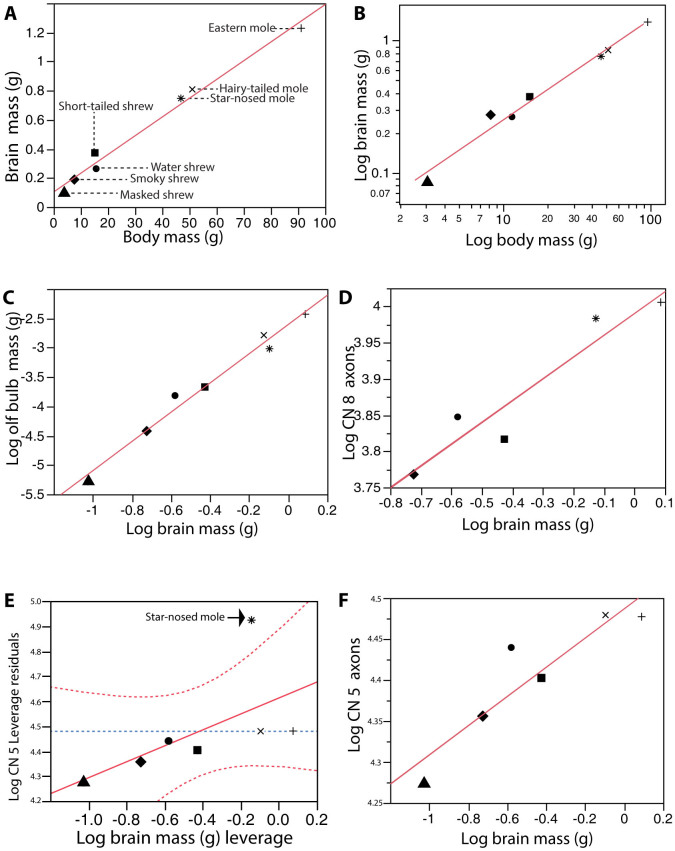
Scaling of brain size, body size, and numbers of myelinated axons within select cranial nerves in seven insectivore species. (A) Scatterplot of brain mass and body mass relationships. Species are indicated. (B) Log-transformed brain mass as a function of log body mass. y = 0.796x − 3.352; *R^2^* = 0.984, p < 0.0001. (C) Olfactory bulb mass (g) as a function of brain mass. y = 2.493x − 2.606, *R^2^* = 0.980, p < 0.0001. (D) Cochlear nerve axons as a function of brain mass. y = 0.300x + 3.990, *R^2^* = 0.906, p = 0.0127. (E) Leverage plot of the residuals of the number of trigeminal axons as a function of brain mass. The average number of trigeminal axons for the star-nosed mole is indicated with the black arrow. (F) Trigeminal nerve axons as a function of brain mass excluding the outlier value from the star-nosed mole. y = 0.179x + 4.486, *R^2^* = 0.845, p = 0.0096. The species symbols in (A) also apply to (B) through (D).

Mammalian brain and body weight data are typically reported on logarithmic scales. We followed this convention to make our data readily comparable to that in similar studies (however, a simple linear model of the untransformed data yielded an *R^2^* of 0.984 for the correlation between body weight and brain weight) (Fig. 3A). We examined regression statistics from both the shrews and moles, and these appeared to fit the conventional allometric equation (log(y) = log (b) + m[log(x)] or y = bx^m^, where *y* is average brain mass and *x* is average body weight. The slope *m* for the brain-body weight regression on log/log scales was 0.796, with *R^2^* of 0.981, p < 0.0001 (Fig. 3B). Furthermore, variance in the logarithm of the mass of olfactory bulbs appeared to be largely explained by changes in either log brain (*R^2^* = 0.980, p < 0.0001) (Fig. 3C) or log body mass (*R^2^* = 0.957, p < 0.0001 plot not shown).

### Optic nerve

**Figure 4:**
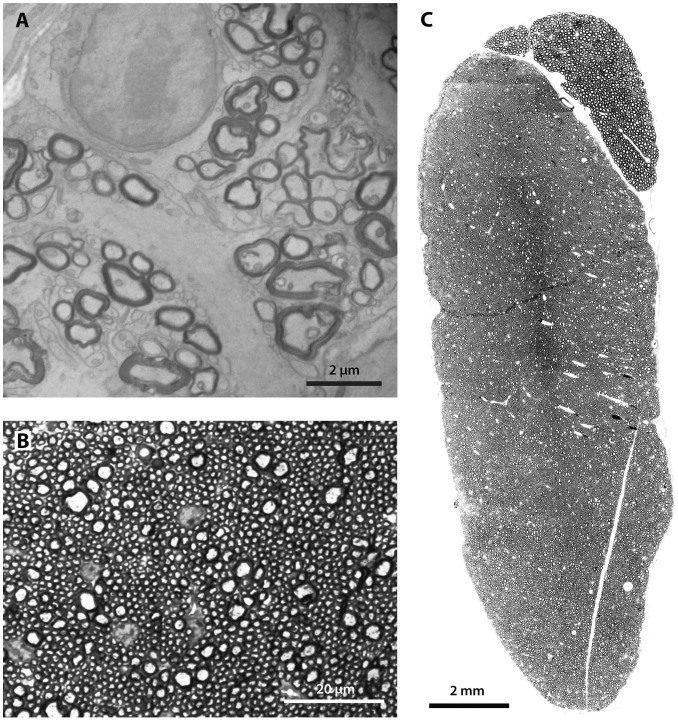
Examples of microscopy of cranial nerves. (A) The optic nerve (CN II), the smallest of those examined, was imaged used transmission electron microscopy to clearly define individual myelinated axons. The example shown is a cross-section from a water shrew. (B) Example of a semi-thin cross-section stained with toluidine blue, as employed in vestibulocochlear and trigeminal nerve preparations. Example shown is a section from a hairy-tailed mole's cochlear nerve (CN VIII). (C) Example of a completed montage of a trigeminal nerve assembled from light microscopy of semi-thin sections as seen in (B). This is a cross-section of the trigeminal nerve (CN V) from the star-nosed mole.

Optic nerve axons were readily distinguished in transverse sections viewed through transmission electron microscopy (TEM). Myelinated fibers were typically 1 to 2 μm in diameter (mean = 1.53; n = 52, Std dev. = 0.39) (Fig. 4A). The layers of the perineurium as well as collagenous connective tissue that ensheathed the entire nerve could be seen in complete TEM montages of each optic nerve. The total number of myelinated axons within the optic nerve showed little relationship to average body (*R^2^* = 0.003, p = 0.914) or brain mass (*R^2^* < 0.0001, p = 0.999) of each species. The fewest optic nerve fibers were noted in the masked shrew (mean = 1483, Std dev. = 115), and the most were found in the water shrew (mean = 6342, Std dev. = 703) (Table S2).

Numbers of myelinated optic nerve axons were compared to log body size and log brain size, but there was no clear relationship for either shrew or mole species.

### Cochlear nerve

The component of the vestibulocochlear nerve exiting the cochlea was sampled approximately 2 to 3 mm from the cochlear bone. Myelinated axons, typically 5–7 μm in diameter, were examined in semi-thin toluidine blue-stained sections under light microscopy (mean = 5.96; n = 63, Std dev. = 0.59) (Fig. 4B). Similar to the optic nerve TEM sections, intact transverse sections of the cochlear nerve were surrounded by perineural sheath. The total number of myelinated axons of the cochlear nerve varied significantly with log brain size (*y* = 0.300x + 3.990, *R^2^* = 0.906, *p* = 0.0127) (Fig. 3D) and log body size across (*y* = 0.240x + 3.553; *R^2^* = 0.963, *p* = 0.0031) across the examined species, with the greatest number of axons found in the Eastern mole (mean = 10,127, Std dev. = 704) and the fewest in the smoky shrew (mean = 5865, Std dev. = 125) (Table S2).

### Trigeminal nerve

The trigeminal nerve was easily distinguished in all dissections as the largest of the cranial nerves. In an identical preparation as was used with the cochlear nerves, trigeminal myelinated axons were viewed in semi-thin sections under light microscopy (Fig. 4C). The larger diameter axons of the presumptive motor component of the trigeminal nerve were visible as a discrete bundle of more thickly-myelinated fibers ensheathed within their own large (1.5–2 mm) fascicle and were excluded from further analyses. The total number of myelinated axons increased with both body mass and brain mass but was not significant (*R^2^* = 0.369, *p* = 0.148 for body and *R^2^* = 0.363, *p* = 0.152 for brain). However the star-nosed mole's trigeminal nerve appeared as an obvious outlier, having many more fibers that any other species in both absolute numbers and relative to its brain and body size. Both the positive leverage residual plot and positive residual value between the data point and the regression confirmed this observation (Fig. 4D). When star-nosed moles were removed from this analysis, the relationship between brain size and trigeminal axon counts was significant (*R^2^* = 0.845, *p* = 0.0096) (Fig. 3E).

## Discussion

The relationship between brain size and body size - and the significance of this ratio for behavior or information processing capabilities - has long been a topic of interest^2^,^23^,^24^. We examine these relationships in seven closely-related Eulipotyphlan species in the shrew and mole genera. These species have a more than 22-fold range in body size, from the miniscule masked shrew to the eastern mole, and also exhibit different peripheral sensory elaborations. Examples of the latter variation can be seen by comparing the relatively sparse mystacial vibrissae of the short-tailed shrew to the elaborate array found in the similarly-sized water shrew (which can use whisker-related tactile cues to locate submerged prey^25^,^26^) or by comparing the thickened, smooth surface of the eastern mole's nose to the vascular, mechanoreceptor-covered nasal rays of the star-nosed mole^27^,^28^,^29^. In addition to providing information about scaling of brains, bodies, and fiber tracts, the measure of afferent fibers provides important insight regarding the sensory priorities and potential sensory abilities in the different species examined.

For example, it is surprising and informative that shrews have very small optic nerves that are essentially the same size or smaller than those of subterranean moles. Indeed, the masked shrew has slightly fewer nerve fibers in its optic nerve (about 1,500) than either the star-nosed mole (1,600) or the hairy-tailed mole (2,600). These numbers are in turn similar to those reported for subterranean mole-rats of various species, which range from 1,000–30,000 optic nerve fibers^30^ for their microphthalmic eyes^31^,^32^,^33^. Indeed, the eyes of the naked mole-rat (*Heterocephalus glaber*) and the blind mole-rat (*Spalax ehrenbergi*) are covered by thick eyelids or skin surfaces^34^. In comparison, similarly sized C57 lab mice have approximately 45,000^35^ to 59,000 myelinated optic nerve fibers^36^. These anatomical observations suggest that both shrews and moles have low visual acuity and probably depend very little on eyesight. Regression analyses showed little correlation between the log of the average brain size and the log of the number of myelinated fibers in the optic nerve in either shrews (*R^2^* = 0.352, *p* = 0.406) or moles (*R^2^* = 0.700, *p* = 0.369). Both groups spend much of their lives in tunnels and grassy runs, apparently requiring less visual information than most terrestrial animals. Both behavioral^26^,^37^ and physiological data from the central nervous system^38^,^39^,^40^ suggest that the visual system of shrews is poorly developed. Often these species forage at night or in low light conditions^41^, situations in which visual information would be less useful. Moles are almost exclusively fossorial, and it is no surprise their optic nerves are small. Even upon careful examination and dissection we failed to find the optic nerves from eastern moles. The small size of their optic nerve may explain their apparent lack of primary visual cortex^42^. Furthermore, among the “microphthalmic” species, it seems unlikely that the scaling relationships present in other mammals reliant on visual cues (e.g., diurnal and nocturnal primates) between their visual systems and brain and body sizes would be conserved^43^. It is also worth noting that the optic nerves of the species examined in this study may represent the lowest limits or “floor” of neural resources for a mammalian visual system, thereby allocating other resources to other sensory systems (i.e., larger trigeminal nerves and CNS representations).

The small size of shrew auditory nerves is also telling in light of the suggestion that some shrews use echolocation^44^,^45^,^46^. It would be surprising if the species examined in the present study had this ability, given their tiny cochlear nerves compared to those of microchiropteran bats, which have 15,000 to 35,000 neurons within the spiral ganglion of the cochlea^47^. Electrophysiological recordings in both mole and shrew species have identified a relatively small area of the caudal portion of the neocortex that appears to responds to auditory cues^42^,^48^, a condition dramatically different from that observed in species known to actively echolocate^49^,^50^.

Finally, star-nosed moles have a trigeminal nerve that was literally off the scale and was therefore removed from the analysis as a statistical outlier. This is a testament to their dependence on information from their elaborate snout and stands out as an example of extreme selection for expanded sensory resolution from a specific sensory surface. Behavioral studies suggest that the expanded trigeminal sensory system in this species facilitates rapid localization and handling of small prey^51^.

The logarithm of brain mass appeared to scale with log body mass, and this relationship was evident from a linear regression (*R^2^* = 0.984, *p* < 0.0001) (Fig. 3). The examined species showed a more than 23-fold difference in body size – from smallest to largest. All Soricid shrew species, ranging in body mass from 3.8 g (*S. cinereus*) to 16 g (*S. palustris*) were smaller than the Talpid moles, which varied from about 50 g (*P. breweri* and *C. cristata*) to 90 g (*S. aquaticus*). Reflecting body size, brain masses were smaller in all shrews compared to the larger moles (Table S1). The olfactory bulb mass showed a 17-fold range among these species. Relative brain mass tended to decrease with increasing body size consistent with the power function with a coefficient of 0.796 (Brain mass = body mass ^0.796^). This suggests that body mass is increasing more quickly than brain mass – a finding that has been repeatedly observed in other insectivores^52^, rodents^53^,^54^,^55^, and a wide range of terrestrial vertebrates^3^,^8^,^56^. Thus, among the shrew and mole species studied, with increasing body size, brains themselves become larger while decreasing in relative mass (brain/body ratio; Table S1.)

In comparisons involving measurements of the overall mass of these structures, it is worth noting the diverse developmental origins of the sensory systems reviewed in this study and the potential relationship between their origins and neural resource allocation. The trigeminal and auditory systems develop from the embryonic neural crest^57^, specifically the trigeminal and otic dorsolateral placodes. However, the olfactory bulb and visual system are part of the formal CNS. The olfactory epithelium is derived from the olfactory/nasal placode, as well. Indeed, the mammalian olfactory bulb has been repeatedly shown to following different scaling constraints than the rest of the brain^16^,^17^, hence the decision to differentiate the olfactory bulb mass from the remaining, non-olfactory CNS mass. However, among the insectivore species studied, olfactory bulb mass appeared to be largely accounted for by body mass or brain mass.

There was little relationship between body size and fiber number in the optic nerve. There is some support for the suggestion that size of the visual system (as part of the CNS proper) might scale in proportion to body size or brain size itself, as predictable relationships have been identified between scaling of individual brain parts, and the entire non-olfactory CNS^16^. Indeed, individual primary sensory areas, including V1, S1, and A1, have been shown to scale with total neocortical areas, and interestingly, the number of rods and cones appears to vary 4–8 fold, even among similarly sized brains (from diurnal and nocturnal mammals)^43^. Although it is possible that the smallest shrews would be challenged to develop and maintain large eyes, given their small head size, the selection pressures that have resulted in such miniscule eyes and optics nerves and the corresponding effect on scaling of the CNS are unknown. However, the number of myelinated axons within the cochlear nerve did scale significantly with increasing brain mass (*R^2^* = 0.906, *p* = 0.013) and body mass (*R^2^* = 0.963, *p* = 0.003). Perhaps this reflects constraints of head size relative to cochlear elaboration, although this is only speculation.

In the case of the trigeminal nerve, as the skin surface becomes larger with increasing body size, it is reasonable to assume that innervating nerve fiber number would follow. In shrews, the trigeminal nerve is largely dedicated to the innervation of the facial vibrissae, whereas in moles, the trigeminal nerve serves both the relatively small whisker pad as well as the mechanosensitive Eimer's organs which cover the glabrous skin of the nose. Moles exhibit a range of Eimer's organ density, with none on the Eastern moles^27^ and approximately 25,000 organs in the facial appendages of star-nosed moles, innervated by a total of more than 100,000 fibers^58^. There was a significant positive relationship between body size and trigeminal afferent number (with outlying star-nosed moles removed from the analysis). In addition to the presumed need for greater fiber number for increasing skin surface area, miniaturization of a sensory sheet could have a similar effect in the opposite direction, calling for reduced fiber number. This is because the number of sensory sinus hairs that can fit on the distal snout of a mammal may decrease at very small sizes. This does appear to be the case, as tiny masked shrews have fewer whiskers than water shrews and short-tailed shrews^48^.

Overall, we found no evidence for differences in brain size correlated with elaboration of the sensory periphery. The most obvious candidate for exhibiting this kind of correlation was the star-nosed mole, with a greatly hypertrophied sense of touch and very large trigeminal nerves containing more than twice the number of fibers found for any of the other species. We considered the hypothesis that selection pressures acting on an magnified somatosensory systems might be manifested in an overall CNS enlargement^16^. However, the star-nosed mole's brain size was not larger relative to its body size compared to the other species examined (Figure 3 A, B). Similarly, the water shrew, with the most elaborate set of whiskers^25^ and largest trigeminal nerve among the shrews examined (Table S2) did not have an unusually large brain size relative to its body size. These results raise the question of what remaining neural structures or cortical areas might be changed in expected size in a push-pull framework for which total size of the CNS is constrained^20^. For example there is some suggestion that trigeminal somatosensory nuclei^14^,^15^ and somatosensory cortex^13^ of the star-nosed mole have reduced whisker representations somewhat “replaced” by the star representation compared to other species^42^. Despite the lack of positive brain scaling relative to afferent number for the trigeminal systems that we investigated, previous studies have revealed other specializations of the central nervous system in these species. For example, the water shrew has a particularly elaborate set of “barrelettes” in the brainstem (modules representing the vibrissae in the trigeminal nuclei)^59^. Similarly, the star-nosed mole has an overall larger principal trigeminal sensory nucleus (PrV) with a large representation of the nose compared to the hairy-tailed mole^14^ and the nasal appendages are well-represented (albeit in different proportions to those found in PrV) in spinal trigeminal subnuclei interpolaris and caudalis^15^. At the cortical level, the star-nosed mole has an elaborate, modular representation of the star that takes up much of somatosensory cortex. Similarly, the whisker representation in water shrews is expansive^40^. Our results suggest that major changes and elaborations of sensory surfaces and fiber tracts are not necessarily accompanied by measurable, corresponding changes in brain size. Such modifications or variation that could result in increasing complexity within the brain (with overall brain size being conserved) include changes to the area of the cortical surface as well as increased modular complexity within specific sensory domains, as noted in primary sensory cortex in a variety of mammals^9^. Indeed, prior investigations that determined neuronal density of the neocortex and subcortical structure among insectivore species suggest that increasing computational power may not be reflected simply in greater neuronal density or overall number of neurons within the neocortex. Increasing evidence suggests that elaboration of a particular sense may be accompanied by addition of cortical areas^60^, magnification of areas of cortical maps^61^, and subdivision of cortical and subcortical regions into separate modules representing specific parts of a receptor sheet^14^,^59^.

## Methods

### Animals

Five masked shrews (*Sorex cinereus*), three smoky shrews (*S. fumeus*), six American water shrews (*S. palustris*), six short-tailed shrews (*Blarina brevicauda*), five hairy-tailed moles (*Parascalops breweri*), and nine star-nosed moles (*Condylura cristata*) were collected using Sherman live traps in Potter and Cameron County, PA., under Pennsylvania Game Commission permits #112-2011 and #COL00087. Six eastern moles (*Scalopus aquaticus*) were captured in Davidson County, TN., under permit Tennessee Wildlife Resources Agency permit #1868. Each animal was weighed and given an overdose of sodium pentobarbital (approximately 200 mg/kg) and then transcardially perfused with phosphate-buffered saline (PBS; pH = 7.4) followed by 4% paraformaldehyde in PBS. The intact brain was dissected from the cranium and weighed. All procedures conformed to the National Institutes of Health standards concerning the use and welfare of experimental animals and were approved by the Vanderbilt University Animal Care and Use Committee (Animal Welfare Assurance Number A-3227-01).

### Cranial nerve preparation

Segments of cranial nerves II, V, and VII (cochlear branch) were dissected approximately 2–5 mm from the point where they became fused with their respective ganglia or CNS nuclei. Optic nerves were sectioned just rostral to the optic chiasm. These tissues were immersed in 2.5% glutaraldehyde solution in PBS for at least 24 hours. They were then post-fixed in osmium tetroxide, dehydrated in a graded ethanol series, placed into propylene oxide, and embedded in EMBed 812 (EM Sciences, Hatfield, PA., USA). For cranial nerve II, ultrathin sections (typically 90 nm) were taken on a Reichert Ultracut E ultramicrotome, mounted on copper mesh grids, stained with uranyl acetate and lead citrate, and imaged on a Philips CM12 TEM (Philips Research, the Netherlands). For cranial nerves V and VIII, semi-thin serial sections (typically 0.5 to 1 μm were cut on an ultramicrotome, stained with 1% toluidine blue, and imaged at 100× on a Zeiss Axioskop (Carl Zeiss Microimaging, Thornwood, NY). Images were compiled in Photoshop CS4 (Adobe Systems Incorporated, San Jose, CA, USA) into complete transverse sections of each cranial nerve, and myelinated axons were manually counted. Whole brains were imaged using a Wild Photomakroskop M400 (Wild Heerbrugg, Gais, Switzerland) with a Zeiss AxioCam HRC (Zeiss, Jena, Germany). Measurements of axon diameter and myelination were performed in ImageJ (National Institutes of Health, Bethesda, MD). Existing data related to total body, brain, and olfactory bulb mass of some insectivores were compiled from the literature and are indicated. All procedures met guidelines set the National Institutes of Health, the Animal Welfare Act, and the Vanderbilt University Institutional Animal Care and Use Committee.

### Statistical analyses

Counts of myelinated axons were averaged for each cranial nerve, and an analysis of variance was performed relative to the brain mass and body mass (and the respective log values of each) to determine whether the slope of the regression curve was significantly different than zero. Outliers for each variable were determined based on analyses of residuals from predicted values and values on leverage “hat” plots. Statistical analyses were performed using the program JMP Version 9.0 (SAS Institute, Cary, NC, USA).

## Author Contributions

D.B.L. and K.C.C. prepared the manuscript. K.C.C. designed the study and analyses. D.B.L. performed measurements of fiber tract size and mass measurements. D.B.L. and D.K.S. collected tissue samples.

## Supplementary Material

Supplementary Information Supplementary Figures 1 and 2
