# Supplementary material for: Brain Mass and Cranial Nerve Size in Shrews and Moles
Source: Sci Rep. 2014 Sep 1;4:6241. doi: 10.1038/srep06241 (PMC4150104; doi:10.1038/srep06241)
Supplement: Supplementary Information — Supplementary Figures 1 and 2 [file srep06241-s1.doc]

Supplementary dataset file to “Brain Mass and Cranial Nerve Size in Shrews and Moles” by Duncan B. Leitch, Diana K. Sarko, and Kenneth C. Catania


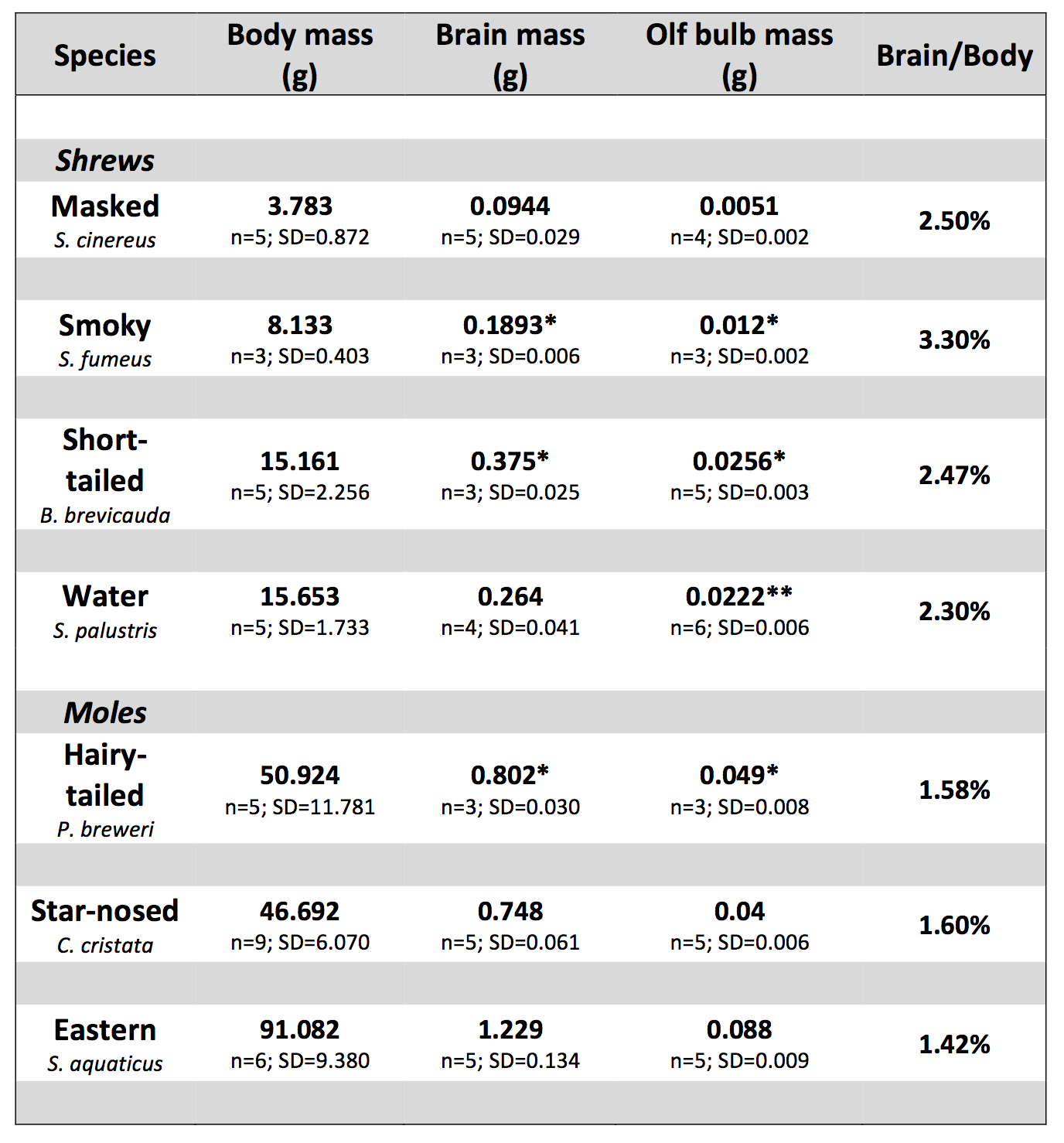


**Table 1.** Measurements of body, total brain, and olfactory bulb mass from shrew and mole species. % indicates the percentage of body mass accounted for by the brain mass. Some data from prior investigations (* from Sarko et al., 2009 and ** from Leitch et al., 2011) were combined with new measurements from this study.


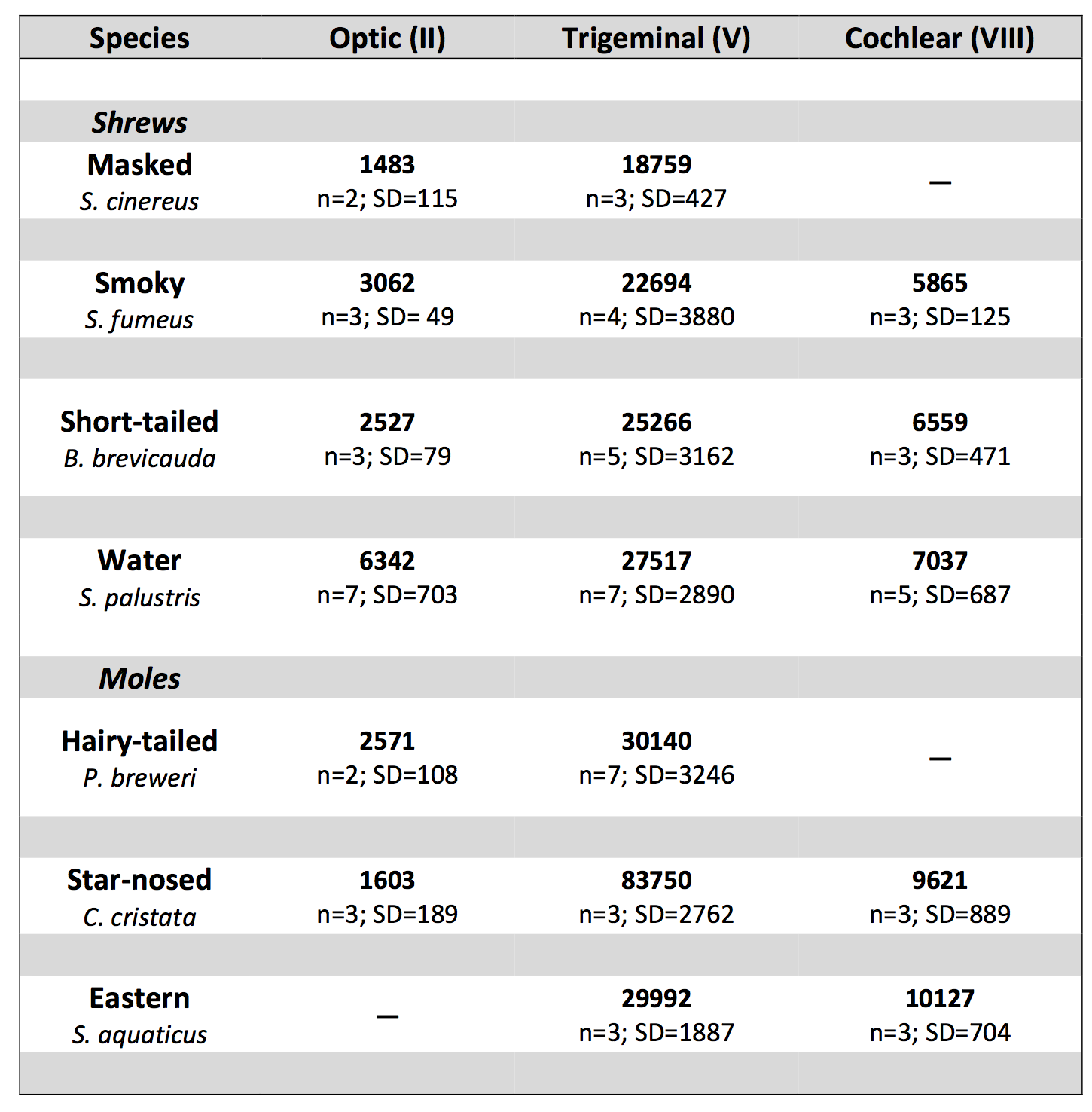


**Table 2.** Quantification of the myelinated axons in the optic, trigeminal, and auditory cranial nerves from 7 shrew species. Below each figure the number of samples counted (n) and the standard deviation (S.D.) are noted.
